# MiniXL: An open-source, large field-of-view epifluorescence miniscope enabling single-cell resolution and multi-region imaging in mice

**DOI:** 10.1126/sciadv.ads4995

**Published:** 2025-06-11

**Authors:** Pingping Zhao, Changliang Guo, Mian Xie, Liangyi Chen, Peyman Golshani, Daniel Aharoni

**Affiliations:** ^1^ Department of Neurology, David Geffen School of Medicine, University of California, Los Angeles, Los Angeles, CA, USA.; ^2^ State Key Laboratory of Membrane Biology, Beijing Key Laboratory of Cardiometabolic Molecular Medicine, Institute of Molecular Medicine, National Biomedical Imaging Center, School of Future Technology, Peking University, Beijing, China.; ^3^ School of Life Sciences, Tsinghua University, Beijing, China.; ^4^ Greater Los Angeles Veteran Affairs Medical Center, Los Angeles, CA, USA.; ^5^ Intellectual and Developmental Disabilities Research Center, University of California, Los Angeles, Los Angeles, CA, USA.

## Abstract

Capturing the intricate dynamics of neural activity in freely behaving animals is essential for understanding the neural mechanisms underpinning specific behaviors. Miniaturized microscopy enables investigators to track population activity at the cellular level, but the field of view (FOV) of these microscopes has often been limited and do not support multi-brain region imaging. To fill this technological gap, we have developed the eXtra Large FOV Miniscope (MiniXL) for mice, a 3.5-gram miniaturized microscope with an FOV measuring 3.5 mm in diameter. We demonstrate the capabilities of the MiniXL through large-scale neuronal population records in hippocampal dorsal CA1. We also demonstrate simultaneous multi-brain region imaging across bilateral medial prefrontal cortex (mPFC) and mPFC and nucleus accumbens (NAc) during complex social behavior and stably track cells across multiple days. As with all microscopes in the UCLA Miniscope ecosystem, the MiniXL is fully open-source and designed to be shared with the neuroscience community to lower the barriers for adoption of this technology.

## Teaser

MiniXL expands open-source neural imaging in freely behaving mice, enabling large-scale, multi-region recordings.

## INTRODUCTION

Imaging of Ca^2+^ indicators (*1*) with head-mounted miniature microscopes (*2*–*6*) has revolutionized the study of neural activity in freely moving animals compared to head-fixation constraints, shedding light on the neural underpinnings of many behaviors such as spatial navigation (*3*), social interaction (*7*, *8*), learning and memory (*9*), sleep (*10*, *11*), and feeding (*12*). While previous iterations of UCLA Miniscopes (*13*–*15*), notably the V4 Miniscope (the newest generation, version 4) (https://github.com/Aharoni-Lab/Miniscope-v4/wiki), and other open-source (*16*–*18*) and commercialized miniaturized microscopes have provided valuable insights by enabling the monitoring of neural dynamics with a field of view (FOV) of up to 1 mm^2^, most of them have been unable to comprehensively map the coordination of neural activity across different brain regions due to their limited FOV.

Previously, we developed an expanded FOV miniaturized microscope called the MiniLFOV (https://github.com/Aharoni-Lab/Miniscope-LFOV) with an FOV of up to 3.6 mm by 2.7 mm and a spatial resolution, in none scattering media, of 2.45 μm (*14*, *19*). However, the MiniLFOV weighed 13.9 g, making it suitable only for rats and larger animals. As mice are the predominant model for circuit-level neuroscience research, there is an urgent need for a lighter and more compact miniaturized microscope with a large FOV, enabling increased cell counts and multi-region imaging of neural activity stably across sessions in freely behaving mice.

In response to this need, here, we introduce the eXtra Large FOV Miniscope (MiniXL), a much lighter version of our head-mounted, open-source, large-FOV miniature microscope as part of the UCLA Miniscope Project. This new model has a 3.5-mm-diameter FOV, with a 4.4-μm lateral resolution, weighs only 3.5 g and is only 30 mm in height. Its front working distance (WD) is electrically adjustable, with a range of 1.9 mm ± 200 μm, facilitated by an electrowetting lens (EWL). Additionally, the MiniXL integrates a nine-axis absolute head-orientation sensor, capable of capturing head movement data at up to 100 Hz, which is instrumental in exploring head orientation–related neural mechanisms.

We tested the MiniXL in freely behaving mice. We imaged GCaMP6f-expressing neurons in the dorsal hippocampal CA1 pyramidal layer in both one-dimensional (1D) and 2D environments, demonstrating robust spatial coding across large population of neurons. Excitingly, we simultaneously measured neural activity in left and right medial prefrontal cortex (mPFC) during a free social interaction task that could be conducted across multiple sessions and days. In addition, we simultaneously imaged neurons in the mPFC and nucleus accumbens (NAc). These experiments confirmed the MiniXL’s stability for long-term cell tracking and capability for multi-region imaging during complex behaviors.

## RESULTS

### System design and optical performance

**Fig. 1.**
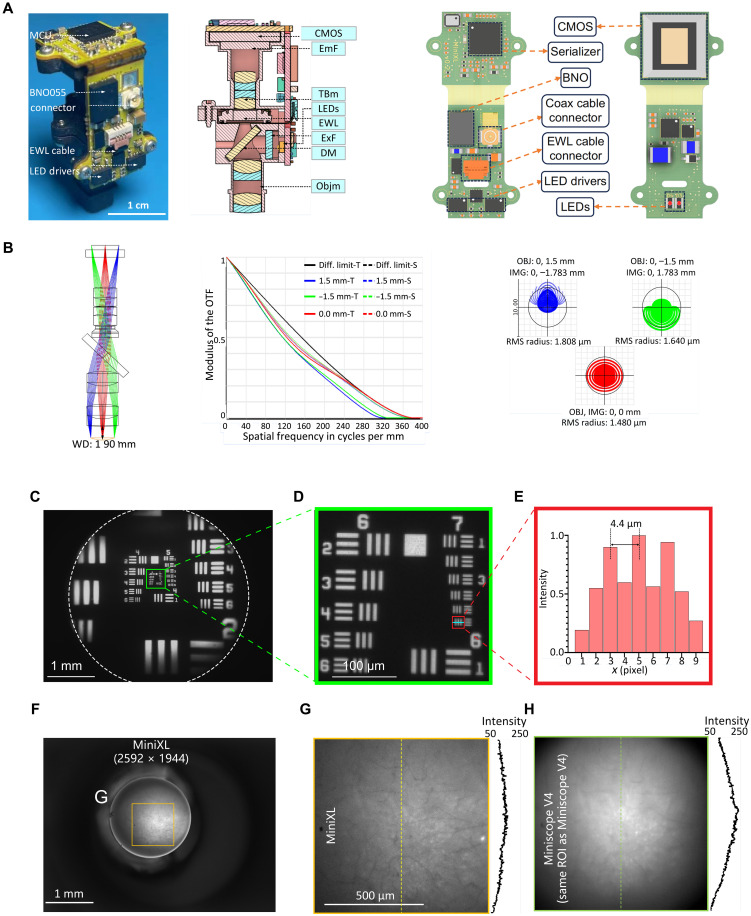
Optical design and performance of MiniXL. (**A**) Left: Photograph of MiniXL. Middle: Cross-sectional profile. Right: PCB layout. Scale bar, 1 cm. CMOS, complementary metal-oxide semiconductor; EmF, emission filter; TBm, tube lens module; ExF, excitation filter; DM, dichroic mirror; Objm, objective module. (**B**) Zemax simulation of emission path shows a 1.9-mm WD with 300-μm field curvature across a 3-mm-diameter FOV in the object space (left). Modulation transfer functions (MTFs) on the image plane (middle), and spot diagram of the optics (right). In the spot diagram, root mean square (RMS) radius is 1.480 μm at the center and 1.808/1.640 μm at (0, 1.5 mm/−1.5 mm). The magnification of the optics is given by 1.19 calculated from the spot diagram. OTF, optical transfer function; T, tangential; S, sagittal; OBJ, objective; IMG, image. (**C** to **E**) FOV (3.5 mm in diameter) shown using the Ø1″ 1951 USAF Target (no. R1DS1N, Thorlabs). Scale bars, 1 mm (C) and 100 μm (D). The value 4.4 μm [group 7 element 6, 228 line pairs (lps)/mm] can be resolved from the green line in (D) and (E). (**F** to **H**) Comparison of MiniXL (F and G) and Miniscope V4 (H) in the same ROI. The FOV of Miniscope V4 is 1.1 mm in diameter. MiniXL shows more uniform fluorescent detection than Miniscope V4. ROI, region of interest.

The MiniXL platform comprises an optics module and a custom rigid-flex printed circuit board (PCB). The PCB integrates several crucial subcircuits: a light-emitting diode (LED) and constant current LED driver for excitation light delivery, EWL and EWL driver for focus adjustment, absolute head-orientation sensor, power distributor, and a complementary metal-oxide semiconductor (CMOS) image sensor for signal capture (Fig. 1A). This assembly is coupled to an optics module containing a quartet of achromatic lenses [nos. 63692, 63691, and 45262 (×2), Edmund Optics], in line with an excitation filter (ET470/40x, Chroma), a dichroic mirror (T495lpxr, Chroma), an emission filter (ET525/50 m, Chroma), and a 3D printed lens holder incorporating an EWL driver (MAX14515, Maxim) for precise focus control. Leveraging a 5-megapixel (MP) monochrome CMOS sensor (MT9P031, Onsemi), the system captures fluorescence signals across a substantial FOV, facilitated by power-over-coax technology and a sophisticated serializer/deserializer pair (DS90UB913A/DS90UB914A, Texas Instruments) based on a single, flexible 50-ohm coaxial cable (CW2040-3650SR, CoonerWire). This arrangement not only ensures streamlined power management and bidirectional communication but also enables high-bandwidth, unidirectional data streaming essential for capturing dynamic neural processes. The system’s compatibility with the UCLA Miniscope Data Acquisition (DAQ) hardware and software extends its functionality, allowing for backward-compatible real-time video streaming and recording, and also enables multi-stream neural and behavioral recording. This DAQ platform supports adjustment of imaging parameters (excitation intensity, EWL focus, image sensor gain, and frame rate), real-time Δ*F*/*F* display, real-time pose estimation (*20*), and synchronization with external devices, enhancing experimental versatility.

Additionally, the system’s numerical aperture (NA) (Fig. 1A) is designed to achieve an optical resolution up to 2.5 μm (NA of 0.12) across a 3.5-mm-diameter FOV by using off-the-shelf lenses. The MiniXL incorporates a Rigid-flex PCB design, comprising four rigid PCBs interconnected by an internal flex-printed circuit. The LED circuit board accommodates two LEDs, which are powered by dedicated LED drivers featuring an Inter-Integrated Circuit (I2C) digital potentiometer for precise excitation light adjustment. Adjacent to the LED assembly, an EWL driver and an EWL cable secure the EWL device to the main PCB board, alongside an absolute orientation sensor (BNO055, Bosch Sensortec) for real-time collection of head orientation data. Additionally, a high-resolution 5-MP monochromatic CMOS image sensor (MT9P031, onsemi) is used to capture Ca^2+^ fluorescence, transmitting digitized image data to the serializer system. This system further processes and serializes the imaging data, transmitting it via a coaxial connector and a single, flexible 50-ohm coaxial cable for seamless integration with a custom Miniscope DAQ system. Its design aims to achieve a balance between resolution, sensitivity, and field coverage while facilitating unconstrained animal behavior, enabling an electrically adjustable WD (1.9 mm ± 200 μm) to accommodate different imaging depths (Fig. 1B). At its core, the system’s 2.2-μm–pixel-size, 5-MP CMOS image sensor expands the achievable FOV while maintaining small form factor optics and cellular resolution (Fig. 1, C to E, and fig. S1). Additionally, the MiniXL has a 9× enlarged FOV, improved optical contrast, and even detection in fluorescence than that of a V4 Miniscope (Fig. 1, F to H, and fig. S2). Higher contrast achieved by the MiniXL is due to multiple factors, including the lower noise characteristics of the improved CMOS image sensor as well as the superior optical performance of the MiniXL’s optics. This is evident in the MiniXL MTF and spot diagrams, which demonstrate better focusing performance than the V4 Miniscope. The MiniXL’s optimized optics, along with its lower NA compared to the V4 Miniscope, contribute to reduced background noise and defocused fluorescence, ultimately leading to higher contrast. The lower NA is more than compensated for by the notably improved CMOS image sensor, further enhancing calcium transient detection and overall image clarity.

The MiniXL weighs 3.5 g, similar in weight to many other existing head-mounted neural recording devices for freely behaving mice (*2*, *13*, *15*, *18*) and does not hinder free behavior (fig. S1). Below, we show that the MiniXL has been successfully used in freely behaving animals across a number of behavioral tasks, including open-field test, linear track, and social interactions paradigms.

### Imaging dCA1 place cells across days

**Fig. 2.**
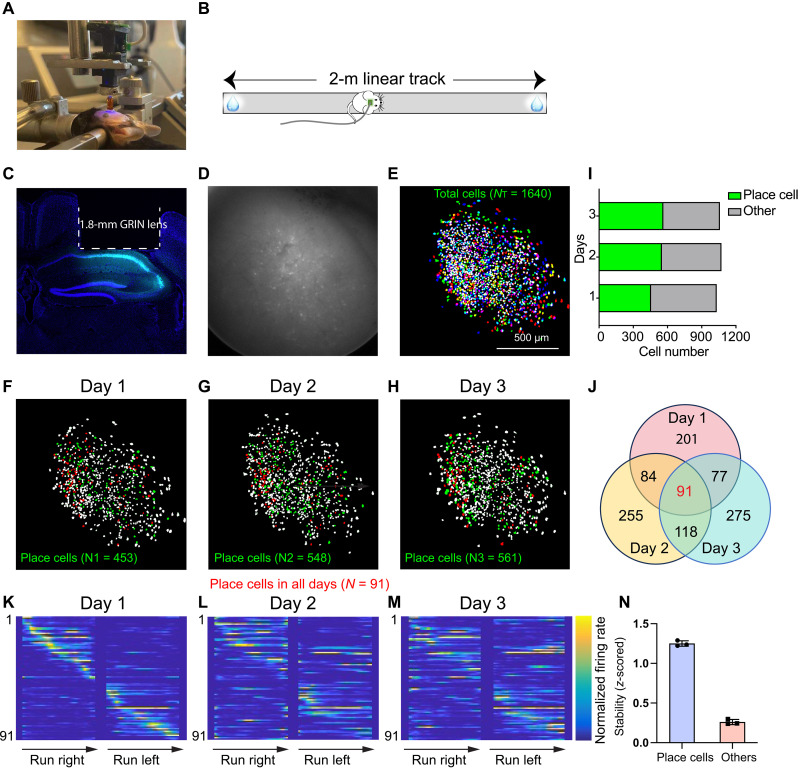
Imaging the activity of dCA1 place cells on a linear track. (**A**) Surgical implantation of GRIN lenses and baseplate. Two GRIN lens were stacked with the bottom one implanted upon dCA1 region. (**B**) Animals were trained to run on a 2-m linear track for water reward on two ends. (**C**) Example brain slice showing GCaMp6f expression and GRIN lens implantation track. (**D** and **E**) Example raw image of FOV and all cells identified across 3 days (*N* = 1640). Each FOV is cropped to ~1.393 mm by 1.393 mm. (**F** to **H**) Identified place cells (green) in one example mouse on day 1 (*N*1 = 453), day 2 (*N*2 = 548), and day 3 (*N*3 = 561). There are 91 cells were identified as place cells and were matched across all sessions (red). (**I**) Ratio of place cells and the other cells on each day. (**J**) Overlap of place cells between every 2 days and among 3 days. (**K** to **M**) Normalized neural activity rates of place cells on day 1 (K) that were also active on day 2 (L) and day 3 (M). cells were sorted by the peak firing rate from day 1. (**N**) Mean stability of place cells and other cells on each day of one example mouse. Place cells showed higher stability than other cells.

To evaluate the MiniXL’s capabilities, we first imaged GCaMP6f-labeled neurons within the dorsal CA1 (dCA1) region, known for its critical role in spatial navigation. Recording place cell activity in CA1 allows us to compare the performance of this microscope with previous versions of miniature microscopes, specifically the UCLA Miniscope versions V3 and V4 and MiniLFOV (*3*, *13*, *14*). We leveraged the large FOV provided by the MiniXL, coupled with implantation of a stacked pair of 1.8-mm-diameter gradient index (GRIN) lenses (Edmund optics, quarter pitch, no. 64-531), resulting in a relay GRIN lens configuration (Fig. 2A). These 1.8-mm– or 2-mm–large-size GRIN lenses have been used frequently for CA1 imaging (*13*, *21*). The incorporation of a large lens and FOV enabled the acquisition of large-scale neural dynamics from 1640 neurons across three sessions (Fig. 2, C to E, and movie S1). Through this setup, we were able to capture the activity of GCaMP6f-expressing neurons in dCA1 as mice navigated a 2-m-long linear track (Fig. 2, B to D). The imaging was conducted at a rate of 23 frames per second (fps) over 15-min intervals, generating high-resolution videos (1000 pixels × 1000 pixels for each frame) that were then cropped to the GRIN lens’s diameter size (1.8 mm in diameter, 704 pixels × 704 pixels) for analysis. Concurrently, a synchronized behavioral camera recorded the mouse’s position at 50 fps, providing a synchronized neural-behavioral dataset for analysis. We used CalmAn (https://github.com/flatironinstitute/CaImAn) and CellReg (*22*), analysis packages that identified, demixed, deconvolved, and tracked 1640 neurons in total across three sessions on different days. A notable proportion of neurons recorded demonstrated spatial tuning in the linear track (44.02% on day 1, 51.21% on day 2, and 53.07% on day 3) (Fig. 2, F to J). The percentage of place cells that we have identified is comparable to that reported in previous studies using miniscope calcium imaging (*13*). This dataset allowed us to identify and track place cells across sessions, showcasing their stability and spatial encoding properties (Fig. 2, K to N).

**Fig. 3.**
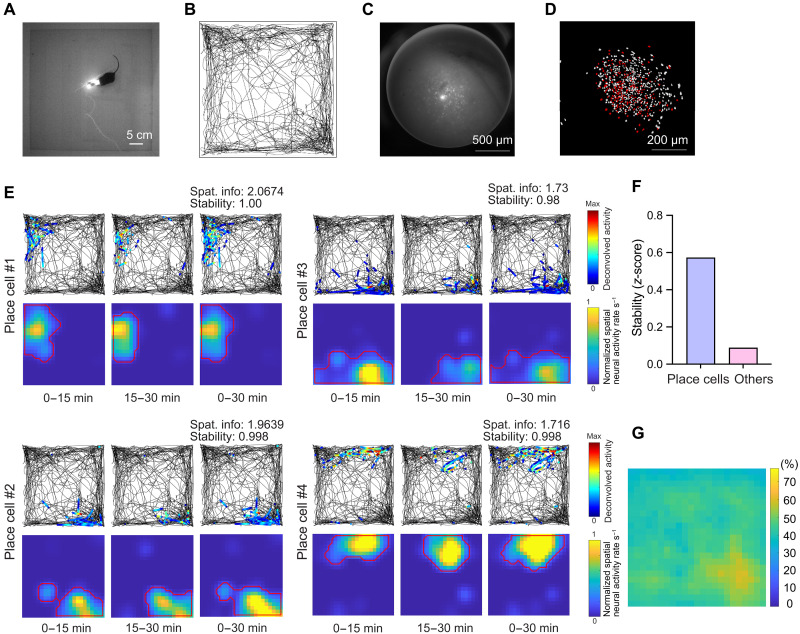
Imaging place cells in the open-field arena. (**A**) Snapshot of mouse behavioral recording in open-field arena. (**B**) The trajectory of the example mouse running in the arena. (**C**) Raw calcium fluorescence of the FOV from example animal. (**D**) Single neurons extracted from the FOV by constrained nonnegative matrix factorization for microendoscopic data with identified place cells indicated in red. (**E**) Example of four places cells with spatial information (Spat. info) value and stability score. (**F**) Comparison of the stability score of place cells and other cells of the example mouse. (**G**) Environment coverage of place fields.

We also tested the MiniXL by recording CA1 neural dynamics during exploration of a 2D open-field arena (Fig. 3, A and B). Across a 30-min recording session, the MiniXL data yielded 764 cells (Fig. 3, C and D), with more than one third of neurons (*N* = 266; 34.82%) meeting the criteria (see Materials and Methods) for place cell identification (Fig. 3D). The analysis highlighted clear spatial preferences and stable activity patterns among identified place cells (Fig. 3, E and F), underscoring the MiniXL’s utility in exploring complex neural phenomena across diverse environment contexts. The red outlined contour denotes the edge of a place field calculated by detecting a 5% cutoff of the binned spatial neural activity rate surrounding the place field. Additionally, the combined open-field place field coverage from all 266 cells fully covered the arena area (Fig. 3G). Mouse speed and time spent in the center of the arena were similar between animals wearing and not wearing a MiniXL (fig. S3, A to C), indicating that MiniXL does not significantly affect locomotion or alter anxiety-related behaviors. This comprehensive approach underscores the MiniXL’s potential in advancing our understanding of neural circuitry and behavior.

### Bilateral imaging of mPFC in freely behaving socially interacting mice

**Fig. 4.**
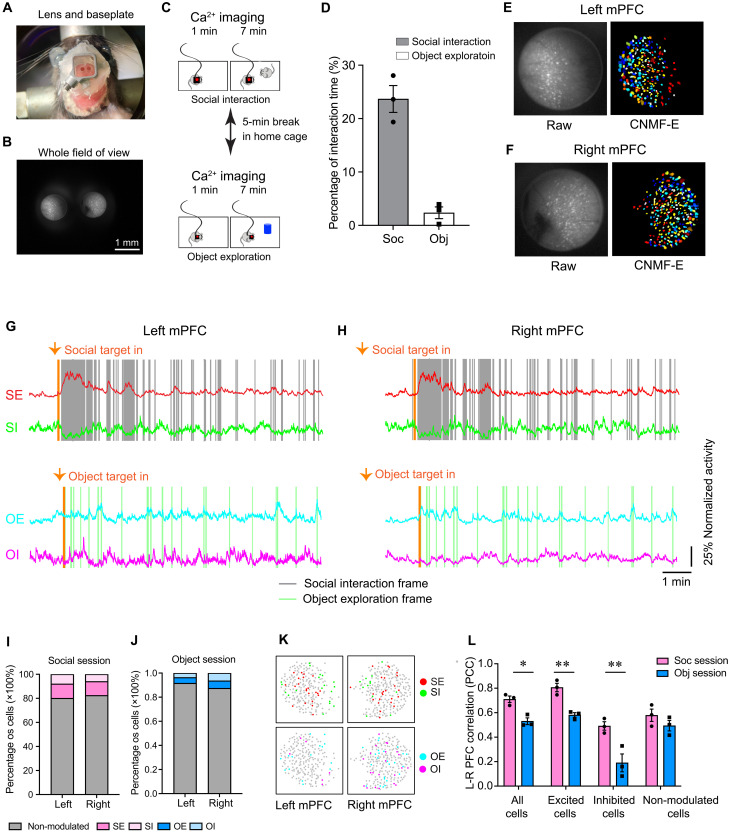
Bilateral imaging of PFC in freely behaving mice using MiniXL. (**A**) Diagram of bilateral lens implantation above left and right mPFC and baseplate installation. (**B**) MiniXL’s FOV across dual GRIN relay lens implants above mPFC. (**C**) Schematic diagram of social interaction session counterbalanced with object exploration during calcium imaging with MiniXL. (**D**) Percentage of time that animal spent on social (Soc) interaction and object (Obj) exploration. (**E** and **F**) Raw calcium fluorescence frame from an example animal and single neurons extracted from the FOV. CNMF-E, constrained nonnegative matrix factorization for microendoscopic data. (**G** and **H**) Mean calcium traces of social excited cells (SE), social inhibited cells (SI), object excited cells (OE), and object inhibited cells (OI) recorded from example mouse’s left and right mPFC. (**I** and **J**) Percentage of SE, SI, OE, OI, and social/object non-modulated cells. *N* = 3 animals. Two-way analysis of variance (ANOVA) followed by Tukey’s multiple comparisons test did not show significant difference between left and right hemispheres’ SE/SI/OE/OI percentage. (**K**) Distribution of SE, SI, OE, and OI in left and right mPFC. (**L**) Comparison of the correlation of left and right (L-R) mPFC during social interaction session and object exploration session. *N* = 3 animals. Two-way ANOVA followed by Fisher’s least significant difference test. All cells, *P* = 0.0173; excited cells, *P* = 0.0057; inhibited cells, *P* = 0.0010; non-modulated cells, *P* = 0.1927. *P < 0.05; **P < 0.01. PCC, Pearson correlation coefficient.

The MiniXL’s large FOV supports simultaneous imaging of more than one relay lens when lenses are located within 3.5-mm distance. Leveraging this ability, we recorded neural activity from bilateral mPFCs during mouse social interactions (Fig. 4C). We implanted two 1-mm-diameter, 4-mm-long relay lenses (Inscopix, 1050-004595) above the right and left prelimbic area of the mPFC labeled with GCaMP6f (Fig. 4, A, B, E, and F, and movie S2) and performed calcium imaging as mice engaged in social or object interactions. As expected, mice engaged more frequently in social interactions (23.7% of total time) compared to object exploration (2.4% of total time) (Fig. 4, C and D). We demonstrated that the lens implantation did not affect animal behavior by comparing social behavior of mice implanted with a single relay lens in the mPFC or not implanted any lens (fig. S4). Moreover, wearing a MiniXL did not significantly affect mouse behavior, including the time spent interacting with social target or the percentage of time spent sniffing, running, rearing, self-grooming, or resting (fig. S3, D to F). Neuronal activity, recorded during these interactions and counterbalanced with object exploration sessions, revealed that a proportion of neurons were significantly activated or inhibited by social or object interaction (Fig. 4, G to J) [left: socially excited neurons (SE), 11.99 ± 0.02%; socially inhibited neurons (SI), 7.92 ± 0.02%; object excited neurons (OE), 4.82 ± 0.01%; and object inhibited neurons (OI) 3.60 ± 0.01%; right: SE, 11.56 ± 0.01%; SI, 5.96 ± 0.02%; OE, 6.22 ± 0.02%; and OI, 6.35 ± 0.02%]. Our findings suggest a similar distribution of neurons responsive to social interactions and object exploration across both hemispheres (Fig. 4K). Notably, the inter-hemispheric neural activity correlation (Pearson correlation coefficient) in the mPFC was higher during social interactions than that in object exploration sessions (Fig. 4L, all cells). Higher correlations were particularly evident among neurons significantly excited or inhibited during social engagement (Fig. 4L, excited cells and inhibited cells), and there was no significant difference during non-interaction period between social session and object session (fig. S5) although no lateralization in social encoding was observed. This underscores a more synchronized neural response in the PFC during social behaviors, highlighting the complex neural coordination underlying social interactions. Moreover, we could track the same cells across multiple days during mice freely social interactions, attesting to the stability of MiniXL imaging (fig. S6).

### Simultaneous imaging of mPFC and NAc core with the MiniXL

**Fig. 5.**
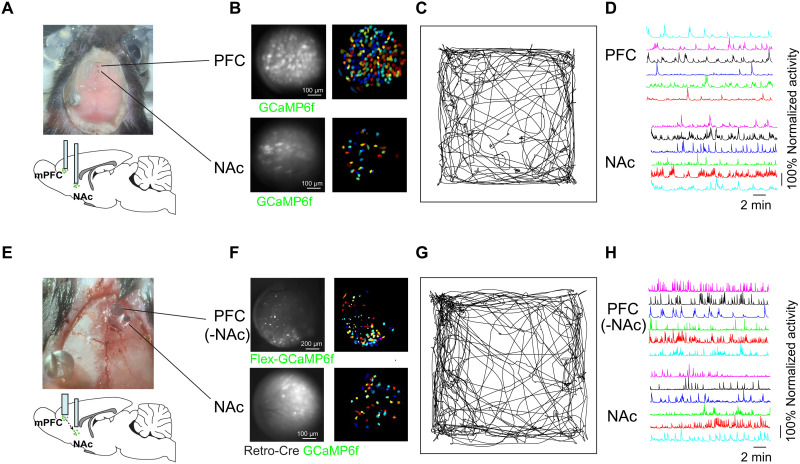
PFC-NAc simultaneous imaging by MiniXL. (**A** and **E**) Diagram of lens implantation in PFC (0.5 mm/6.1 mm in A and 1 mm/4 mm in E) and NAc (0.5 mm/8.4 mm). (A) NAc, GCaMP6f; PFC, GCaMP6f. (E) NAc, retro-Cre and GCaMP6f; PFC, Flex-GCaMP6f. (**B** and **F**) Raw calcium fluorescence frame of the FOV from example animals and single neurons extracted from the FOV. (**C** and **G**) Trajectory of mouse in open-field test. (**D** and **H**) Example of calcium traces recorded from NAc and mPFC.

Understanding the neural dynamics driving behavior requires measuring coordinated activity patterns across multiple brain regions within a network. To highlight this capability, we performed simultaneous imaging of mPFC and NAc core during an open-field test (Fig. 5), demonstrating the technical feasibility of dual-region imaging. Neurons within both the mPFC and NAc core were labeled with GCaMP6f. This was achieved through the careful choice of relay lenses: a 0.5-mm-diameter, 6.1-mm-long lens (Inscopix, 1050-004599) for the mPFC, and a 0.5-mm-diameter, 8.4-mm-long lens (Inscopix, 1050-004600) for the NAc, ensuring comprehensive coverage of the targeted areas (Fig. 5, A, B, and D, and movie S3).

Given the known direct projections between the PFC and NAc, which encodes a blend of social and spatial information (*23*), we adopted a specific genetic labeling strategy. This involved the injection of a retrograde AAV expressing Cre recombinase into NAc, combined with injections of AAV-Syn-Flex-GCaMP6f in the mPFC (Fig. 5, E to H). Because of the topological distribution of mPFC neurons projecting to different downstream target, we implanted 1-mm relay lens above mPFC to increase the chance of recording from target cells. This approach selectively labels mPFC neurons projecting to the NAc, illuminating the upstream-downstream dynamics within this circuit. Using the MiniXL, we successfully imaged the activity of mPFC neurons projecting to NAc simultaneously with the activity of NAc neurons themselves. Combined with different virus strategies, this dual-region imaging technique could become a versatile tool for exploring neural circuits of various behavior and enrich our understanding of underlying neural mechanisms.

## DISCUSSION

The MiniXL marks a substantial advance in miniaturized microscopy imaging technology, offering several key enhancements over existing miniature microscope platforms such as the UCLA Miniscope versions V3 and V4 (table S1), as well as the TINIscope (*16*), the Kiloscope (*17*), and CM^2^ (*24*). Its design enables imaging from a large FOV (3.5 mm diameter), at cellular resolution (4.4 μm) at 23 fps while maintaining a low weight (3.5 g). The adjustable WD of 1.9 mm ± 200 μm and a novel absolute head-orientation sensor further increase the flexibility and capability of the microscope. The previous released Kiloscope also provides cellular resolution imaging across a large FOV and an even lighter weight. However, the aspheric lens assembly (originally used for smartphone) and the optical fiber results in difficulty assembly and less flexible tethering during behavior. All parts for a MiniXL are off-the shelf products and are easy to assemble. The MiniXL’s tether, a single coaxial cable with 0.3 mm in diameter, supports a wider range of natural behavior and also makes it backward compatible with all UCLA Miniscope DAQs. This innovative platform facilitates diverse imaging modalities, enabling both deep and superficial brain region long-term imaging.

Through in vivo testing, we have validated the MiniXL’s capability to capture neuronal activity across various brain regions, including the dCA1 of the hippocampus, bilateral mPFC, and mPFC with NAc simultaneously. Complementary to previous studies showing persistent imaging of a large number of bilateral hippocampal neurons (*25*), a single MiniXL has the ability to simultaneous record more than 500 neurons from unilateral dCA1 or nearly 500 neurons from bilateral mPFC in freely behaving mice within a single session, expanding the population recorded and allowing for more comprehensive studies of neural population dynamics across multiple brain regions including symmetric brain regions in the left and right hemispheres and brain regions inside one functional system such as mesolimbic reward system (fig. S7).

Furthermore, the MiniXL, supported by an array of online training resources and documentation provided by the UCLA Miniscope project (https://github.com/Aharoni-Lab), presents an open-source, user-friendly, and cost-effective solution for the neuroscience community. This accessibility encourages the broader adoption of calcium imaging techniques in research laboratories, fostering further discoveries in animal behaviors and neural function while minimizing technical and economic barriers.

The MiniXL also sets a new standard for multi-region brain imaging, essential for understanding the complex interactions within neural circuits that underpin behavior. Its dual-region imaging capability, highlighted here by simultaneous imaging of the mPFC and NAc, addresses a critical need for tools that can explore the neural basis of behaviors coordinated by multiple brain regions. This feature is particularly relevant for studies on social behavior, learning and memory, and so on, where understanding the interplay between different areas of the brain and the information flow in the network is the key to unraveling the mechanisms of social interaction.

In comparison to other devices like the TINIscope and Kiloscope, the MiniXL stands out for its single-coaxial-cable tethering and electronic focal plane adjustment, which simplifies the experimental setup and minimizes interference with natural animal behaviors. Additionally, the MiniXL is backward compatible with the UCLA Miniscope DAQ hardware and software as well as can be adopted to use the UCLA Miniscope’s optional wire-free expansion module for rats and larger animals (*14*). Its high frame rate and large FOV make it an excellent fit for studies requiring detailed imaging of complex neural networks. UCLA Miniscope project has released supportive information as usual to assist users in adopting the technology (Github: https://github.com/Aharoni-Lab/MiniXL). In conclusion, the MiniXL represents an important open-source advance in in vivo neural imaging technology.

## MATERIALS AND METHODS

### MiniXL design, manufacturing, and assembly

MiniXL was designed using Zemax Optics Studio. The NA was set to be 0.12 in the objective space and FOV was set at a minimum of 3 mm diameter. The distance between chosen lenses were optimized to achieve <4-μm resolution across at least 3-mm FOV while maintaining the weight of scope <4 g for mice. Two achromatic lenses (nos. 63-692 and 63-691, Edmund Optics) were used to build the 1.9-mm-WD objective module. Two achromatic lens (no. 45-262, Edmund Optics) were adopted to build the emission module. The EWL (Corning Arctic 25N, Varioptics/Corning) is placed between the objective and emission modules for electronic focus adjustment. The MiniXL bodies, including objective module, emission module, filter cover, and baseplate, were designed in Autodesk Fusion 360 (educational license) and printed with black resin (FLGPBK04, Formlabs) by a stereolithographic 3D printer (Form 3, Formlabs). Three filter slots for an excitation filter (4 mm by 4 mm by 1.1 mm; ET470/40x, Chroma), a dichroic filter (4 mm by 6 mm by 1 mm; T495lpxr, Chroma) and an emission filter (5 mm by 5 mm by 1 mm; ET525/50m, Chroma) were designed on the microscope bodies. All the optical components could be easily assembled without the need for any epoxy or optical glue. The circuit schematic and rigid-flex PCB layout were designed using KiCad, a free software suite for electronic design automation. The PCB was divided up into two rigid subcircuits. One was an excitation circuit including LEDs, an EWL tuning, and head orientation chip. The other was a CMOS image sensor circuit with a serializer chip. The two subcircuits were connected by a double-sided embedded flex printed circuit. The assembly of all the components uses seven M1 thread-forming screws (96817a704, McMaster-Carr).

### Animals

All experimental protocols were approved by the Chancellor’s Animal Research Committee of the University of California, Los Angeles, in accordance with the National Institutes of Health guidelines. All mice used here were C57BL6/J mice obtained from the Jackson Laboratory (strain 000664). Mice were older than 6 weeks old. They were maintained on a 12-hour:12-hour light/dark cycle with food and water ad libitum (except the mice doing linear track). Mice were single housed after surgery for 3 to 4 weeks before in vivo calcium imaging and behavior experiments. Except that fig. S3 includes both male and female mice, other data have been collected from male mice.

### Stereotaxic surgeries

Mice were anaesthetized with 1 to 2% isoflurane-oxygen mixture and placed into a stereotactic frame (David Kopf Instruments). AAV1.Syn.GCaMP6f.WPRE.SV40 was unilaterally injected into dCA1 (400 nl) [anterior-posterior (AP), −2.1 mm; medial-lateral (ML), 2 mm; dorsal-ventral (DV), −1.65 mm], bilaterally injected into mPFC (prelimbic cortex) (300 nl each) (AP, +1.8 mm; ML, 0.4 mm; DV, −2.1 mm), or injected into mPFC (400 nl) (AP, +1.9 mm; ML, 0.5 mm; DV, −2.5 mm) and NAc (400 nl) (AP, +1.3 mm; ML, 1 mm; DV, −4.7 mm) of 6- to 7-week-old mice at 60 nl min^−1^ using a Nanoject microinjector (Drummond Scientific). Alternatively, retro-Cre (400 nl) and Syn.GCaMP6f (350 nl) were injected into NAc, and Flex-GCaMP6f (400 nl) was injected into mPFC. Five to 7 days after virus injection, mice were implanted with a 1.8-mm-diameter, 4.7-mm-long GRIN lens (Edmund Optics) over dCA1(coordinates of lens center: AP, −2.1 mm; ML, 1.8 mm; DV, −1.25 mm) or 1-mm-diameter, 4-mm-long relay lens (Inscopix) over mPFC in each hemisphere (coordinates of lens center: mPFC: AP, +1.8 mm; ML, 0.5 mm; DV, −1.8 mm); 0.5-mm-diameter, 6.1-mm-long relay lens in mPFC and 0.5-mm-diameter, 8.4-mm-long relay lens in NAc (coordinates of lens center: mPFC: AP, +1.9 mm; ML, +0.5 mm; DV, −2.5 mm; NAc: AP, +1.3 mm; ML, 0.75 mm; DV, −4.3 mm); and 1-mm-diameter, 4-mm-long relay lens in mPFC and 0.5-mm-diameter, 6.1-mm-long relay lens in NAc (coordinates of lens center: PFC: AP, +1.9 mm; ML, +0.5 mm; DV, −2.5 mm; NAc: AP, +1.3 mm; ML, +1 mm; DV, −4.7 mm). Lenses were secured to the skull using cyanoacrylate glue and dental cement and covered with Kwik-Sil (World Precision Instruments). Two to 3 weeks later, MiniXL was attached to an aluminum baseplate and placed on top of the GRIN lens. After adjusting the focal plane, we secured the baseplate with dental cement. Lenses were protected by plastic cup over baseplate.

### Behavioral tests

#### Open-field test

Mice with GRIN lenses implanted in dCA1 or mPFC and NAc were habituated to the environment and to wearing MiniXL for 6 days before test. On days 1 and 2, mice wore dummy scopes weighing 3.5 g in the home cages for 1 hour. On days 3 to 6, mice wore dummy scopes in the home cages for 30 min and in an open arena (45 cm by 45 cm by 30 cm) with visual cues on the walls for 30 min. On the day of test, the microscope was attached to the baseplate, and we recorded calcium signals and behavioral videos simultaneously when mice are freely moving in the arena.

#### Linear track

After finishing open field, mice were water restricted and maintained at a body weight of around 85% of their initial weight. Mice were trained to run back and forth along the track wearing dummy scopes with similar weight as real scopes while experimenters pipetted 10 μl of water reward at each end of the track. Mice were habituated to the track for 15 min each day for 3 days. After habituation, imaging session was performed every 2 days for three sessions. Calcium imaging was performed simultaneously with MiniCAM imaging of mouse behavior at 50 Hz.

#### Social interaction

Mice with bilateral relay lens implantations were habituated to the environment and to wearing dummy MiniXL for 4 days before testing. On the days of test, mice were habituated for 30 to 60 min in their home cage inside the experimental room. During the first session on day 1, a MiniXL was attached to the baseplate, and the mouse was placed into an open arena (45 cm by 45 cm by 30 cm). After the subject mouse explored the environment alone for 1 min, a novel C57BL/6 male mouse was introduced into the arena as a social target. The two mice interacted freely for 7 min. The subject mouse was then put back into the home cage alone for 5 min, after which it was transferred back to the same arena. After 1 min being alone in the arena, a 3D printed object (10-cm-diameter, 10-cm-high cone) was placed in the center of the arena as an object target that the subject mouse explored for 7 min. Calcium imaging was performed simultaneously with behavioral recording by a MiniCAM both at 22 Hz.

### Analysis of behavioral assays

#### Linear track and open field

For experiments involving freely behaving mice, behavioral videos were captured in AVI format using an overhead MiniCAM (50 fps). The position of the mouse was extracted by tracking the position of the blue light from the excitation LED. The pixel location of the LED was found by detecting the highest pixel value region within the grayscale behavior recording frames, and then the pixel value location is converted to real-world coordinates.

#### Social interaction

Animal’s behavior was recorded by MiniCAM from the top of arena at 22 Hz. All videos were manually annotated frame by frame to identify the onset and offset of each epoch of social interaction and object exploration. The following criteria must be met for an interaction to be identified as a social or object interaction episode: (i) The subject mouse (mouse wearing miniXL) must initiate investigation of the social target (or object) or respond to the exploration by social target mouse. (ii) Social interaction must include approaching, sniffing, grooming, chasing, and mounting the social target. The onset of interaction was identified as the frame in which subject nose pointed to the social (object) target within a distance about ^1^/_4_ to ^1^/_3_ of its body length. The offset of interaction was identified as the frame in which subject nose started to leave the social (object) target at a distance about ^1^/_4_ to ^1^/_3_ of its body length.

#### Calcium imaging analysis

Calcium imaging data were analyzed using MiniscopeAnalysis package (https://github.com/etterguillaume/MiniscopeAnalysis) or CalmAn (https://github.com/flatironinstitute/CaImAn-MATLAB) (*26*). The NoRMCorre algorithm was applied to perform motion correction (*27*). The constrained nonnegative matrix factorization for microendoscopic data approach was used to identify and extract the spatial shapes and fluorescent calcium activity of individual cells (*28*). All cells’ shape and Ca^2+^ traces were manually inspected to ensure high data quality. For imaging data recorded from sessions across days, we applied CellReg package (https://github.com/zivlab/CellReg) (*22*) to identify the same cells across days.

### Identifying place cells

#### Place cells in linear track

For a particular cell to be identified as a place cell in recordings of mice running on the linear track, it must meet the following three criteria: (i) The neuron’s spatial information content must exceed chance levels (*P* < 0.05) as determined by circularly shuffled distribution analysis. (ii) The neuron’s within-session stability (both the first and second half sessions) must exceed chance levels (*P* < 0.05) as determined by circularly shuffled distribution analysis. (iii) The spatial activity rate map of the neuron must demonstrate activity in consecutive bins covering a minimum of 10 cm, with activity rates ranking in at least the 95th percentile among the binned activity rates of circular trial shuffled spatial activity rate maps (*13*).

The information content was defined as

$$\begin{array}{c} I=\sum_{i=1}^{N}p_{i}\frac{\lambda_{i}}{\bar{\lambda }}{\text{log}}_{2}\frac{\lambda_{i}}{\bar{\lambda }},p_{i}=\frac{t_{i}}{\sum_{i=1}^{N}t_{i}},\bar{\lambda }\;=\;\sum_{i=1}^{N}p_{i}\lambda_{i} \end{array}$$

where $t_{i}$ represents the occupancy time spent in the *i*th bin and $\lambda_{i}$ represents the neural activity rate in the *i*th bin. The stability was calculated by taking the Fisher *z*-score of the Pearson correlation coefficient between the spatial activity rate maps at two time points (odd versus even trials and first half versus second half of the trials).

Spatial neural activity rates were calculated using 2-cm-wide spatial bins and a speed threshold of greater than 10 cm s^−1^. Temporal neural activity and occupancy of the animal were spatially binned and then smoothed using a Gaussian kernel with σ = 5 cm. The binned neural activity was divided by the binned occupancy to calculate the spatial neural activity rate of each cell.

#### Place cells in open-field arena

To identify place cells in mice exploring a 2D environment, the following criteria needed to be met: The spatial information content need to be significantly above chance (*P* < 0.05) based on the circularly shuffled distribution; the neuron’s within-session stability both the first and second half sessions) must have exceeded chance levels (*P* < 0.05); and the spatial activity rate map of the neuron must have covered five adjacent bins (*14*).

### Identifying social cells/object cells

Imaging data and behavior videos were synchronized using timestamps derived from the computer’s internal clock. For each cell, calcium traces were normalized to the maximum value in the entire recording session. We used receiver operating characteristic (ROC) analysis (*29*, *30*) to identify social interaction responsive cells and object exploration responsive cells. For each cell, we calculated an area under ROC curve (auROC) value (0 to 1) to measure the overlap of calcium traces with binarized social and object interaction plots and compared these values to those obtained from 1000 times randomly shuffled traces. Neurons with auROC values over 97.5 percentile of shuffled data were classified as social excited cells (SE) or object excited cells (OE). Neurons with auROC values below the 2.5 percentile of shuffle data were classified as social inhibited cells (SI) or object inhibited cells (OI). The remaining neurons were classified as non-modulated neurons (SN and ON).

### Statistics

All statistical analyses were conducted using GraphPad Prism 9 and MATLAB (R2020a). All plots were presented in means ± SEM unless otherwise specified. Statistical significance was defined with *P* < 0.05. All statistical methods and sample numbers are described in individual figure legends.
