# Supplementary material for: MiniXL: An open-source, large field-of-view epifluorescence miniscope enabling single-cell resolution and multi-region imaging in mice
Source: Sci Adv. 2025 Jun 11;11(24):eads4995. doi: 10.1126/sciadv.ads4995 (PMC12154187; doi:10.1126/sciadv.ads4995)
Supplement: Supplementary file 1 — Figs. S1 to S7 Table S1 Legends for movies S1 to S3 [file sciadv.ads4995_sm.pdf]

Science Advances
AAAS

Supplementary Materials for

MiniXL: An open-source, large field-of-view epifluorescence miniscope enabling single-cell resolution and multi-region imaging in mice

Pingping Zhao *et al.*

Corresponding author: Daniel Aharoni, daharoni@mednet.ucla.edu; Peyman Golshani, pgolshani@mednet.ucla.edu

Sci. Adv. **11**, eads4995 (2025)
DOI: 10.1126/sciadv.ads4995

The PDF file includes:

Figs. S1 to S7
Table S1
Legends for movies S1 to S3

Other Supplementary Material for this manuscript includes the following:

Movies S1 to S3

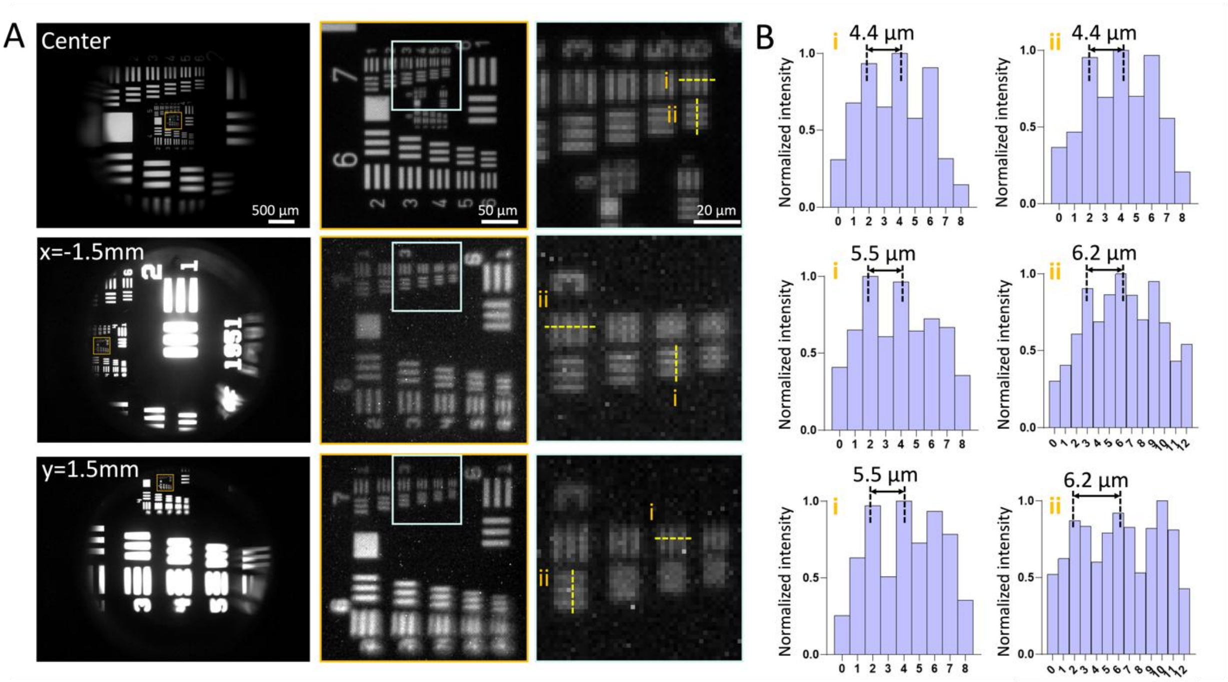

Fig. S1 Resolution across the field of view. (A) The finest strips in the resolution target are located in the center of FOV, 1.5mm offset in X direction and 1.5mm offset in Y direction respectively. The same excitation intensity is used for the zoomed-in regions for comparison. (B) The resolution is 4.4 µm (group 7 element 6, 228 lps/mm) in the center, while decreasing to 6.2 µm (group 7 element 3, 161 lps/mm) around the 3mm FOV.

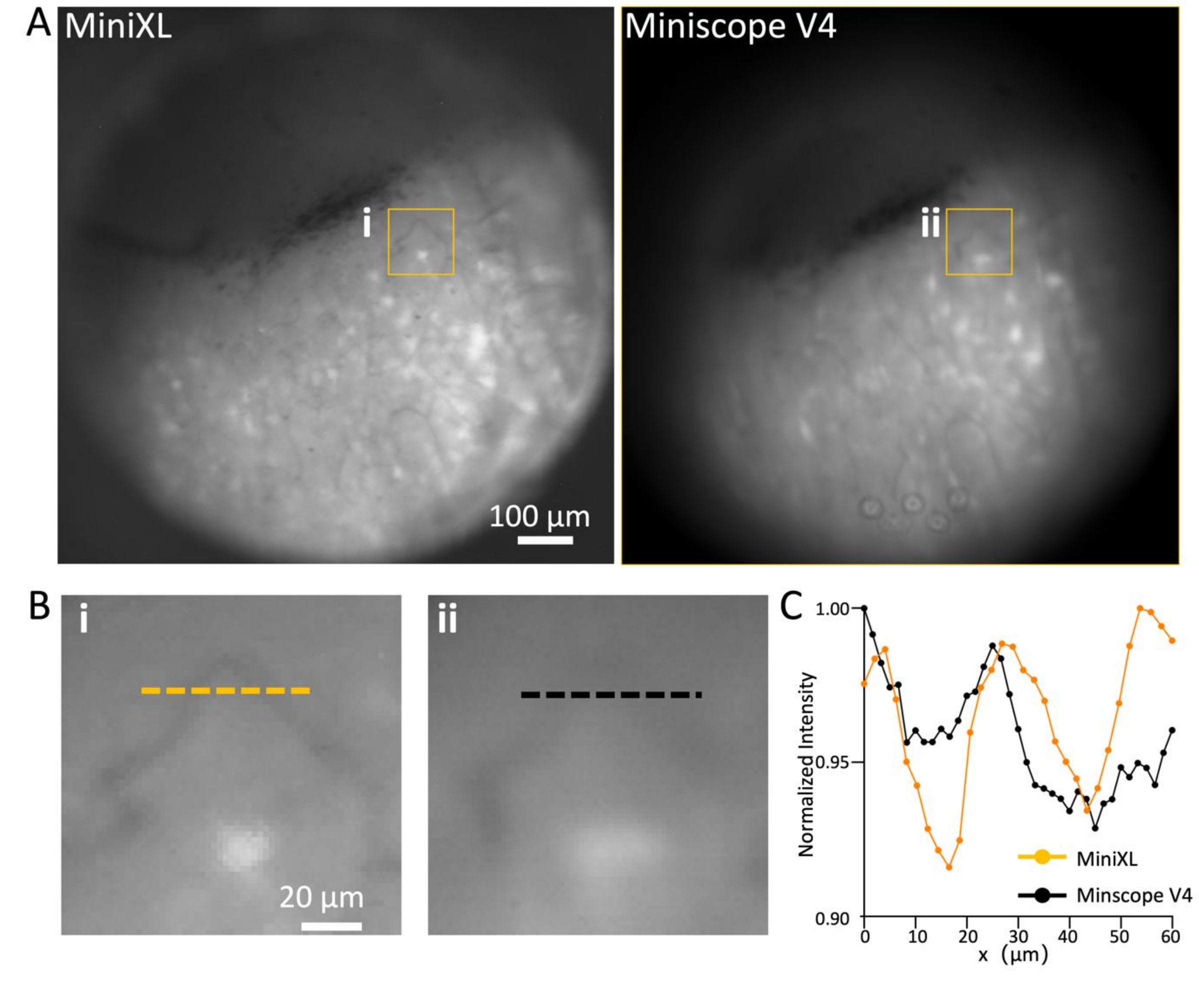

Fig. S2 Contrast comparison of MiniXL and Miniscope V4. (A) Average of 50s recording of MiniXL (left) and Miniscope V4 (right). (B) Zoomed-in regions in MiniXL and Miniscope V4. (C) The profile of the blood vessels in B, in which the MiniXL shows better contrast compared to Miniscope V4.

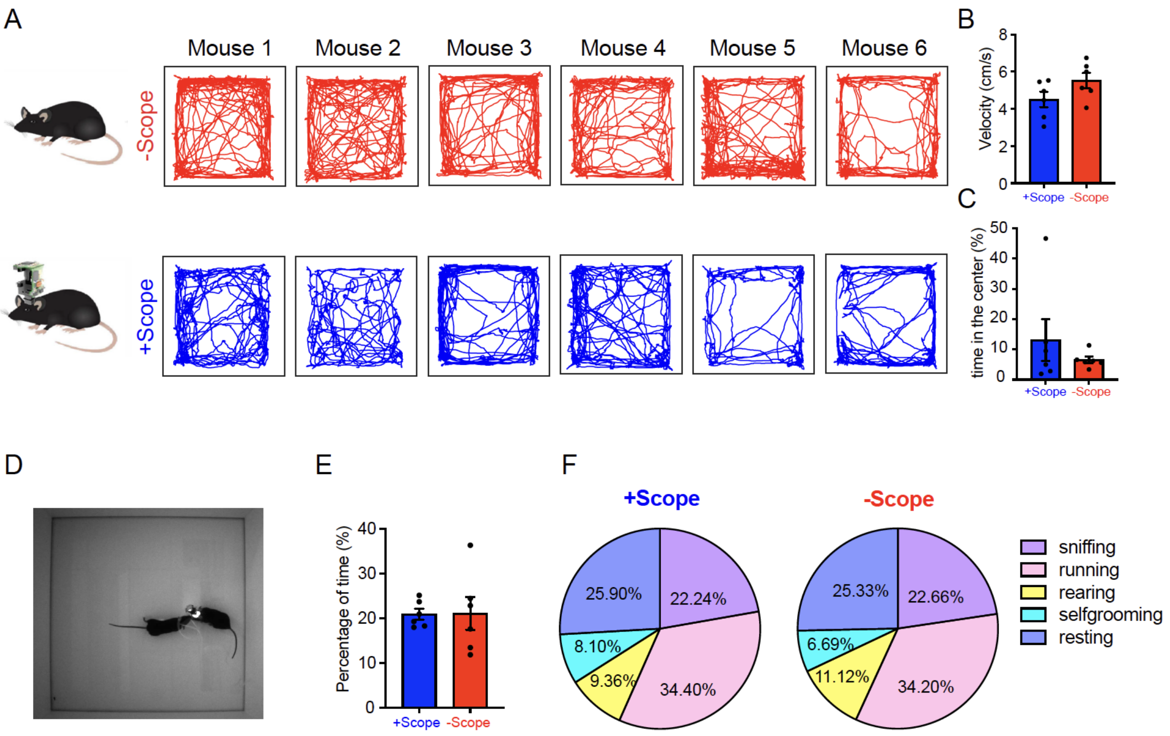

Fig. S3 No significant difference detected in open field test and social interaction test with MiniXL. (A) Schematic diagram of mouse inside open field area with visual cues and trajectories of six mice with or without wearing MiniXL. (B) Velocity of mice travelling in the arena with or without wearing MiniXL. Wilcoxon matched-pairs singed rank test, P=0.4375. (C) Percentage of time mice spent in the center area of the chamber with or without MiniXL. Wilcoxon matched-pairs singed rank test, P=0.5625. (D) A snapshot of mice social interaction test. (E) Percentage of time subject mice interacting with social target with or without MiniXL. Wilcoxon matched-pairs singed rank test, P=0.8438. (F) Pie chart showed percentage of different types of natural behavior subject mice performed. Two-way ANOVA followed by Šídák's multiple comparisons test, P=0.9992, no difference between +Scope group and -Scope group. N=6 animals.

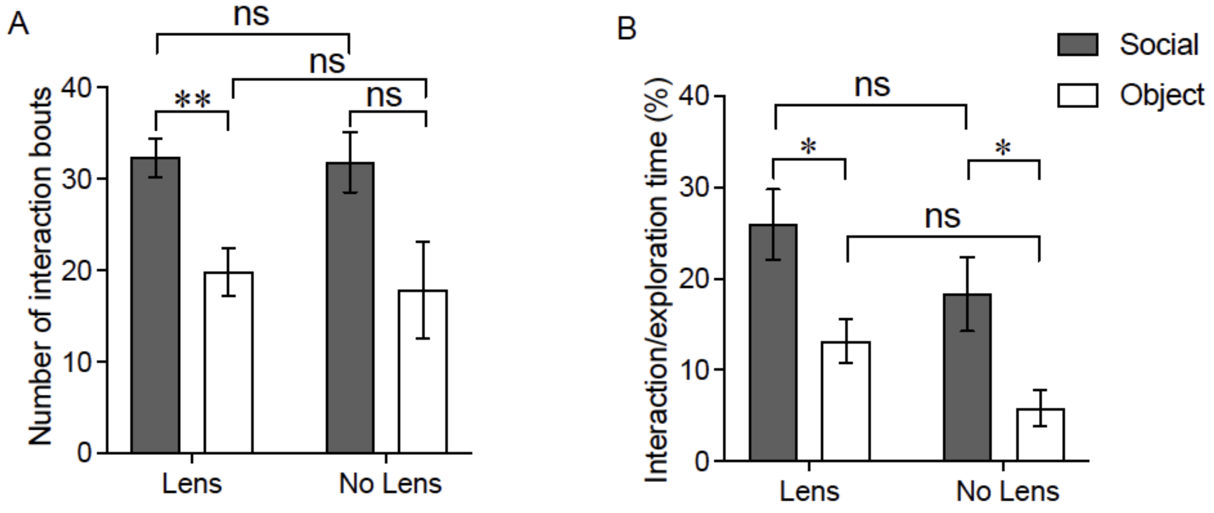

Fig. S4 Lens implantation does not change mice social behavior. (A) Number of interaction bouts initiated by subject mice to social target and object. Use Mann Whitney test for comparison between Lens group and No lens group. Use Wilcoxon matched-pairs signed rank test for comparison between social session and object session within Lens group or No lens group. In Lens group, Social vs Object, P=0.0068. (B) Percentage of time mice spent on social interaction and object exploration. With lens implantation, N=11, Without lens implantation, N=6. Use Mann Whitney test for comparison between Lens group and No lens group. Use Wilcoxon matched-pairs signed rank test for comparison between social session and object session within Lens group or No lens group. In Lens group, Social vs Object, P=0.0186. In No lens group, Social vs Object, P=0.0312. * P<0.05, ** P<0.05. Mice had lens implanted into NAc and wearing V3 or V4 miniscope.

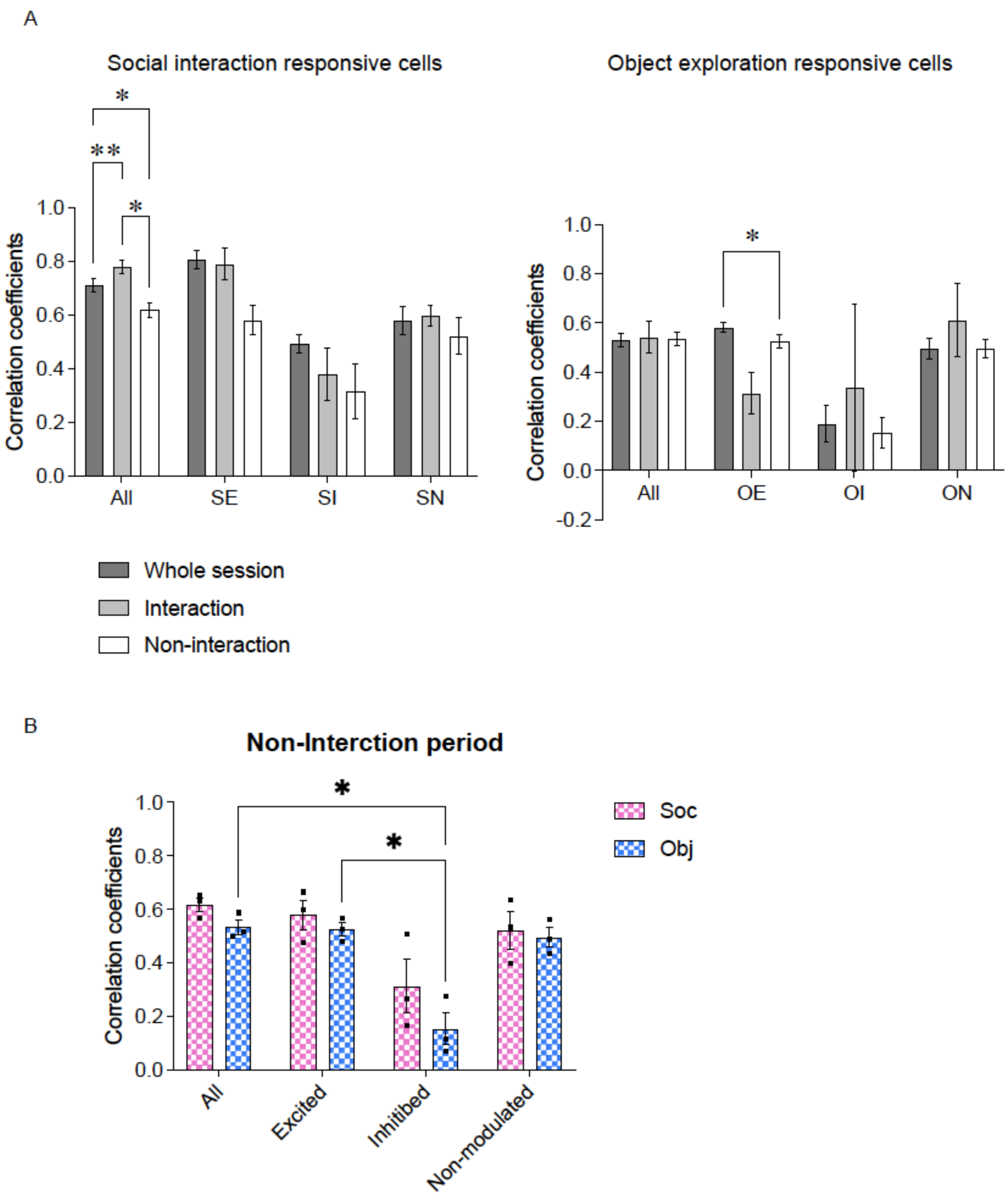

Fig. S5 Correlation coefficients between left and right mPFC neurons. (A) Correlation coefficients of left and right mPFC neurons during social interaction sessions (left panel) and object exploration session (right panel). SE, social excited cells; SI, social inhibited cells; SN, non-modulated cells; OE, object excited cells; OI, object inhibited cells; ON, object non-modulated cells. Two-way ANOVA followed by Fisher's LSD test. Left panel, all cells, whole session vs interaction period, P=0.0085;

whle session vs non-interaction period, P=0.0214; interaction period vs non-interacion period, P=0.0131. Right panel, Object excited cells, whole session vs non-interaction session, P=0.026. (B) Correlation coefficients of left and right mPFC neurons during a non-interaction period of social session and object session (white bars in A). Two-way ANOVA followed by Fisher's LSD test. Object session, all cells vs object inhibited cells, P=0.0407; object excited cells vs object inhibited cells, P=0.0258. N=3 animals.
*P<0.05, **P<0.01.

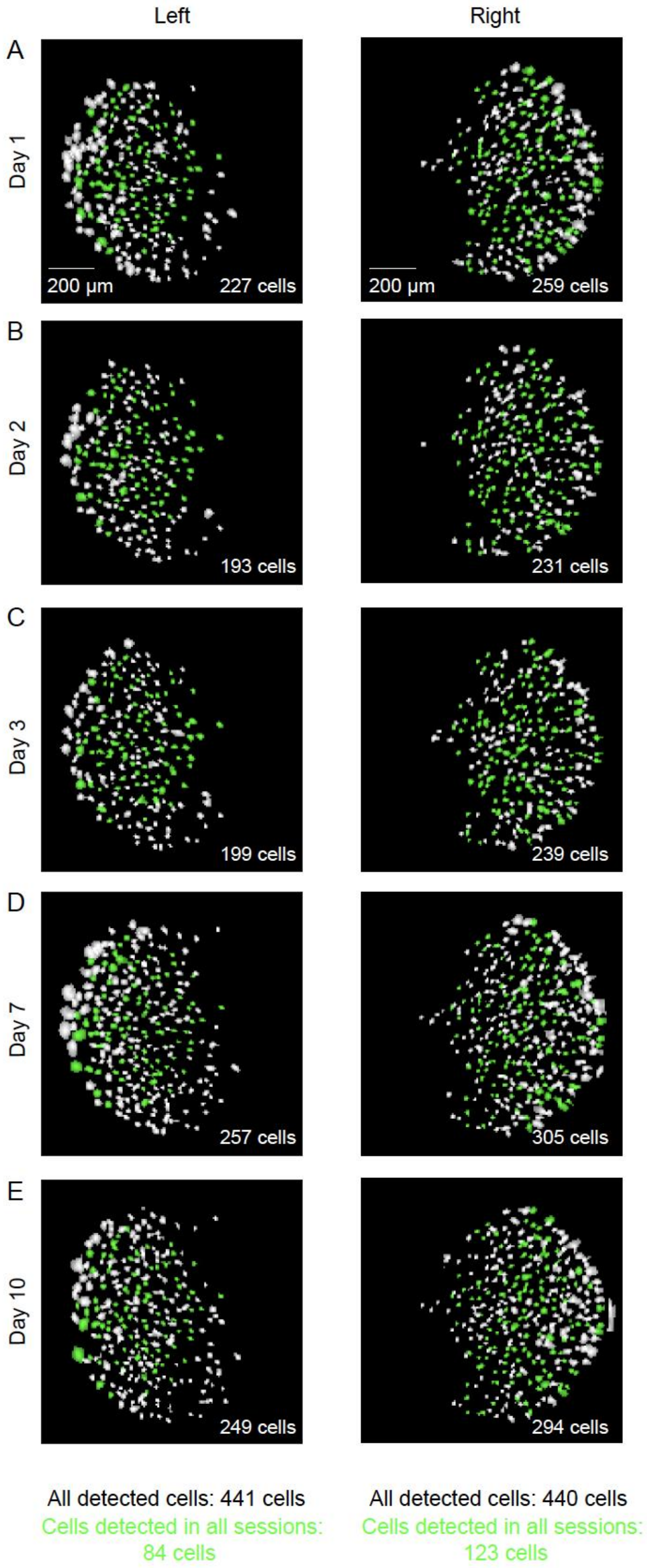
Left
Right
A
Day 1
200 μm
227 cells
200 μm
259 cells
B
Day 2
193 cells
231 cells
C
Day 3
199 cells
239 cells
D
Day 7
257 cells
305 cells
E
Day 10
249 cells
294 cells
All detected cells: 441 cells
Cells detected in all sessions: 84 cells
All detected cells: 440 cells
Cells detected in all sessions: 123 cells

Fig. S6 Bilateral imaging of PFC in freely social interacting mice across multiple days using MiniXL.

(A-E) CNMF-E extracted cells from left and right mPFC on different days. Green labelled cells are those detected from all sessions.

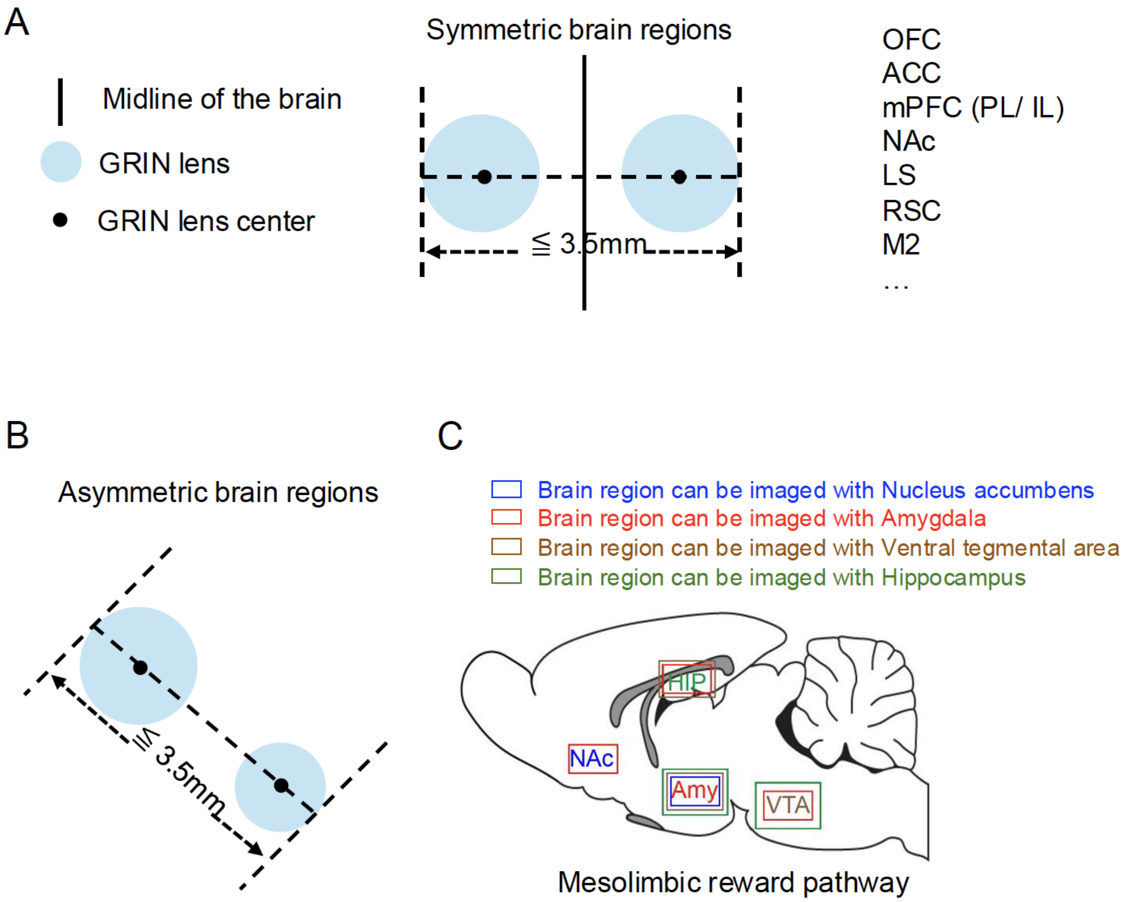

Fig. S7 Potential permutations of brain regions that can be imaged simultaneously with MiniXL.

(A) Example symmetric brain regions in the left and right hemispheres can be imaged simultaneously using MiniXL.

(B and C) Example combination of brain regions in mesolimbic reward pathway can be imaged simultaneously using MiniXL. Lenses sizes can be different depend on the experimental design.

Table S1. UCLA Miniscope ecosystem.

**UCLA Miniscope ecosystem**	Miniscope V3	Miniscope V4	MiniLFOV	MiniXL
Weight	3.3g	2.5g	13.9g	3.5g
Dimensions	16.5 X 13 X 22.5 mm	15.6 X 16.9 X 22.3 mm	35 mm tall (L-shape)	15 X 15 X 30 mm
Electronic focus	No	Yes	Yes	Yes
wire-free capability	Yes	Yes	Yes	Yes
FOV	700 X 450 µm	1000 µm in diameter	3.6 X 2.7 mm	3.5 mm in diameter
Resolution	≤4.4 µm	4.4 µm	2.5 µm	4.4 µm
Maximum frame rate (Hz)	30 Hz	120 Hz	30 Hz (2X2 binning)	30 Hz (2X2 binning)

Movie S1. Raw video of Calcium signals recorded from dCA1 when mouse running on the linear track.

Movie S2. Raw video of Calcium signals recorded from bilateral mPFCs during mouse social interaction test.

Movie S3. Calcium signals recorded from NAc and PFC simultaneously synchronized with animal behavior during open field test.
Upper left: screen shot of cropped field of view (left, NAc; right, mPFC).
Upper middle and Right: motion corrected videos of NAc and mPFC calcium signals.
Bottom: Trajectory of the mouse running in the chamber.
